# The Process of Producing Bioethanol from Delignified Cellulose Isolated from Plants of the Miscanthus Genus

**DOI:** 10.3390/bioengineering7020061

**Published:** 2020-06-21

**Authors:** Olga Kriger, Ekaterina Budenkova, Olga Babich, Stanislav Suhih, Nikolay Patyukov, Yakov Masyutin, Vyacheslav Dolganuk, Evgeny Chupakhin

**Affiliations:** 1 School of Life Science, Immanuel Kant Baltic Federal University, Nevskogo 14, Kaliningrad 236006, Russia; olgakriger58@mail.ru (O.K.); kbudenkova@gmail.com (E.B.); olich.43@mail.ru (O.B.); stas-asp@mail.ru (S.S.); sved_08_89@mail.ru (N.P.); yma1989@mail.ru (Y.M.); 2 Kemerovo Institute of Food Science and Technology, Kemerovo State University, Krasnaya 6, Kemerovo 650043, Russia; dolganuk_vf@mail.ru

**Keywords:** *Miscanthus*, delignification, cellulose fermentation, ethanol, microorganism consortium, biofuel

## Abstract

Plants of the Miscanthus genus (*Miscanthus* Anderss.) have a unique index of biomass production in relation to the occupied area. Miscanthus plants can be attributed to promising second-generation raw materials for the production of bioethanol and biofuel. Miscanthus plants are characterized by a high cellulose content. Herein, we report the results of a study on the obtained delignified cellulose with subsequent processing into bioethanol using microbial communities. In the course of the study, the optimal conditions for the delignification of the initial plant material for cellulose were selected. Ethanol with a high degree of conversion was successfully obtained from the isolated delignified cellulose. The article describes the pilot technological scheme for the conversion of Miscanthus plant biomass to bioethanol involving the delignification stages, followed by the conversion of the resulting cellulose into bioethanol by a consortium of microorganisms. As a result of the study, it was found that delignification using trifluoroacetic acid leads to the production of cellulose of high purity. Bioethanol with a yield of 3.1% to 3.4% in terms of the initial amount of biomass was successfully obtained by a microorganism consortium of *Saccharomyces cerevisiae* *M Y-4242/Pachysolen tannophilus Y-3269*, and *Scheffersomyces stipitis Y-3264*.

## 1. Introduction

Miscanthus is a fast-growing C4 rhizomatous grass. This plant requires a small amount of water or fertilization and can be harvested every year with high biomass yields. As a promising energy crop, Miscanthus spp. have a high yield of biomass per unit area. Particularly, a biomass yield up to 41 t/ha/y can be achieved for some *M. sinensis* hybrids [1].

Pretreatment of Miscanthus with hot water or alkaline results in a significant release of glucose; thus, the glucose yields can be 90% or higher in the case of pretreatment such as ammonia fiber expansion (AFEX) that combines both chemical and physical processes [2]. Ethanol is produced by yeast fermentation of the hydrolyzate derived from the enzymatic hydrolysis of the residual solids (pulp) after pretreatment. Calculations suggest that simultaneous saccharification and fermentation of cellulose furnish 0.13 to 0.15 g ethanol/g-raw biomass [3].

Bioethanol derived from Miscanthus is a high value added product [4]. The production of ethanol requires a lignocellulosic biomass of Miscanthus to be pretreated and enzymatically hydrolyzed to produce fermentable sugars for the microbial fermentation. There is a wide range of chemical pretreatment methods that employ acids, alkalis, alcohols, organic acids, pH-controlled liquid hot water (LHW), or ionic liquids. From the aforementioned approaches, acid hydrolysis using diluted sulfuric acid (H2SO4) is the most common option. As a result of the diluted acid pretreatment, carbohydrates (mainly hemicelluloses) depolymerize to oligo- and monosaccharides. The latter process is accompanied by side products formation, including furfural, acetic acid, and 5-hydroxymethyfurfural (HMF) that are toxic for the following fermentation [5]. To avoid the contamination of the resulting mixtures with undesired hydrolysis products, alternative methods for the delignification of Miscanthus are being actively developed. Specifically, recent reports describe the use of formic/acetic acid [6], microwave heating [7,8], and hydrogen peroxide/formic acid [9] as pretreatment agents. Despite these facts, the described protocols for the production of ethanol from different Miscanthus spp. mostly employ dilute sulfuric acid delignification.

Another factor that strongly affects the efficiency of the whole process is the choice of microbial consortium for the cellulose fermentation. Currently, the following microorganisms are widely used, *Candida shehatae, Pichia* (*Scheffersomyces*) *stipitis*, and *Pachysolen tannophilus* [10], providing various yields of the target product as well as different levels of tolerance toward the presence of the lignification side products. Thus, the efficiency of the delignification and fermentation process parameters is crucial for the efficiency of bioethanol production. 

In the present study, we report a novel protocol for the production of ethanol from Miscanthus Zebris. The study involved the following steps: (1) selection of laboratory parameters for the process of plant Miscanthus biomass delignification, (2) study of the technological characteristics of the obtained delignified cellulose, (3) investigation of the enzymatic hydrolysis of the obtained cellulose, and (4) study of the process for producing bioethanol from a fermented cellulose hydrolyzate by a consortium of microorganisms. 

## 2. Materials and Methods

All chemical reagents were purchased from Sigma-Aldrich: sodium hydroxide, acetic acid, trifluoroacetic acid (TFA), benzoic acid, hydrogen peroxide, sulfuric acid, acetonitrile, α-cyano-4-hydroxycinnamic acid, dinitrosalicylic acid (DNS), (NH2)2SO4, KH2PO4, MgSO4·7H2O, NH4Cl, K2HPO4, peptone, and maltose with reagent grade to the highest degree of purity. Water MQ grade was prepared in a purification system. The following species and strains of microorganisms were used to select a consortium of microorganisms: *Aspergillus niger* F-1270, *Coprinus delicates* F-248, *Kluyveromyces Marxianus* Y-2039, *Kluyveromyces Marxianus* Y-2137, *Saccharomyces Stipites* Y-3263, *Saccharomyces Stipites* Y-3264, *P. Tannophilus* Y-2246, *Pachysolen Tannophilus* Y-3269, *Pachysolen Tannophilus* Y-3270, *Saccharomyces cerevisiae Q* Y-4245, *Saccharomyces cerevisiae M* Y-4242, *Saccharomyces cerevisiae W* Y-4246, *Saccharomyces cerevisiae N* Y-4243, *Saccharomyces ludwigii 8* Y-2012, Zygo*saccharomyces Rouxii* Y-4659, *Saccharmyces Bacillaris* Y-4015, *Lacahncea Thermotolerans* Y-4532, and *Tarulaspora Delbrueckii* Y-1539. All microorganisms were obtained from the collection of Federal Institution “State Research Institute of Genetics and Selection of Industrial Microorganisms of the National Research Center”, Kurchatov Institute, Russia, Moscow.

### 2.1. Miscanthus Plant Collection

*Miscanthus sinensis* Zebrinus plants were harvested during active growth during the summer. To obtain delignified cellulose, stems and aerial parts of the plant were used. The stems were pre-dried and ground in a laboratory mill. Plants were harvested during the period of active growth in the Krasnodar Territory of the Russian Federation. The collection was conducted from June to August 2019. Whole parts of the plants were milled using the laboratory mill Grindo MIX 200 by Retsch. The resulting mass after grinding was sieved through a sieve with a mesh diameter of 1 mm. The resulting particles were dried to constant weight as described below. The result was an average sample weighing 500 g.

### 2.2. Drying Plant Materials and Determination of Chemical Composition

The aboveground parts of plants and stems were dried in an oven at a temperature of 90 °C to constant weight. Crude protein (8.8 ± 0.2%), fiber (42.24 ± 1.2%), and moisture (12.7 ± 0.5%) were determined for a medium sample [11].

### 2.3. Methods of Delignification

The delignification process was carried out according to general methods. The effectiveness of the delignification process was controlled by the molecular weight profile of the extracted lignins using the MALDI-TOF (matrix-assisted laser desorption/ionization with time of flight mass spectrometry) technique. MALDI-TOF analysis for lignin quality control and delignification efficiency was carried out according to [12].

#### 2.3.1. Method A

An amount of 10 g of dry Miscanthus plant was placed into a round-bottom flask equipped with a magnetic stirrer, reflux condenser, and thermometer. Amounts of 100 mL of water and 4 g of NaOH were added to Miscanthus plant. The reaction mixture was stirred for 3 h at 75 °C. The reaction mixture was cooled to room temperature and colorless cellulose fibers were filtered-off. The chemical composition of the filtrate was evaluated by MALDI-TOF spectroscopy.

#### 2.3.2. Method B

An amount of 10 g of dry Miscanthus plant was placed into a round-bottom flask equipped with a magnetic stirrer, reflux condenser, and thermometer. Amounts of 50 mL of water, 30 mL of acetic acid, 20 mL of 30% hydrogen peroxide, and 2 mL of sulfuric acid were added to Miscanthus plant. The reaction mixture was stirred for 3 h at 75 °C. The reaction mixture was cooled to room temperature and colorless cellulose fibers were filtered-off. The chemical composition of the filtrate was evaluated by MALDI-TOF spectroscopy.

#### 2.3.3. Method C

An amount of 10 g of dry Miscanthus plant was placed into a round-bottom flask equipped with a magnetic stirrer, reflux condenser, and thermometer. Amounts of 50 mL of water, 30 g of benzoic acid, 20 mL of 30% hydrogen peroxide, and 2 mL of sulfuric acid were added to Miscanthus plant. Reaction mixture was stirred 3 h at 75 °C. The reaction mixture was cooled to room temperature and colorless cellulose fibers were filtered-off. The chemical composition of the filtrate was evaluated by MALDI-TOF spectroscopy.

#### 2.3.4. Method D

An amount of 10 g of dry Miscanthus plant was placed into a round-bottom flask equipped with a magnetic stirrer, reflux condenser, and thermometer. Amounts of 50 mL of water, 30 mL of trifluoroacetic acid, 20 mL of 30% hydrogen peroxide, and 2 mL sulfuric acid were added to Miscanthus plant. The reaction mixture was stirred for 1 h at 60 °C. The reaction mixture was cooled to room temperature and colorless cellulose fibers were filtered-off. The chemical composition of the filtrate was evaluated by MALDI-TOF spectroscopy.

#### 2.3.5. MALDI—TOF Spectroscopy

All mass spectra were recorded using a Bruker Autoflex time-of-flight mass spectrometer with an ionization system using laser ionization desorption in the linear mode of scanning positively charged ions. The settings in all cases were as follows: the voltage at ion sources was 19.5 and 18.45 kV, respectively, the voltage of the ion lens was 8 kV; laser: Nd: YAG, 1 GHz emission frequency, 500 pulses per measurement. In all cases, a saturated solution of α-cyano-4-hydroxycinnamic acid in a 1:1 mixture of acetonitrile/water with the addition of 0.1% trifluoroacetic acid was used as a matrix. The measurements were carried out in three parallels. The spectra were obtained by summing the spectra for the three measurements.

#### 2.3.6. Hydrolysis of Cellulose by Microorganisms

Microbial consortia for cellulose fermentation were obtained as described in [13]. The strains of microscopic fungi *Aspergillus niger* F-1270 and *Coprinus delicatulus* F-248 were cultured on solid nutrient medium (g/L: maltose—30, peptone—1, agar—20). After the second passage, a suspension in a culture medium was prepared from one colony of the strain (g/L: maltose—30, peptone—1). The biocompatibility tests of the strains were carried out in a suspension with an optical density of 0.10–0.11. The biocompatibility test of strains of microscopic fungi *Aspergillus niger* F-1270 and *Coprinus delicatulus* F-248 was performed out according to the direct co-cultivation method [14,15]. In this case, a drop of a suspension of one strain of microorganisms was placed on the surface of a solid nutrient medium; after drying, the drops were placed next to a drop of the other test strain in such a way that they overlapped by half. Petri dishes were incubated for 24 h at a temperature of 25 °C.

To carry out the enzymatic hydrolysis of a substrate, the sample was suspended in an acetate buffer (0.1 g/mL, pH 5.0). The suspension was inoculated with a concentration of 0.60–0.61, and the optical density amounted to 5% of the total sample volume. In the case of introducing microorganisms into the sample of the consortium, the total volume of the inoculum was divided between the components of the consortium. Enzymatic hydrolysis of cellulose was carried out in an incubator shaker at 30 °C and 100 rpm for 5 days. Samples for the photometric analysis of completeness of cellulose hydrolysis were taken once a day at regular intervals.

#### 2.3.7. Reducing Sugar Content Measure after Cellulose Fermentation

The completeness of hydrolysis was measured by the yield of reducing sugars (RS) [16]. In a 25 mL volumetric flask, 1 mL of the solution was added after cellulose fermentation, and intensively mixed with 2 mL of DNS. The flask was placed into a steam bath and boiled for 5 min. After cooling the reaction mixture, the absorption was measured at 530 nm using a UV-3600 Schimadzu spectrophotometer (see Supplementary Materials). The measurements were carried out in three parallels.

#### 2.3.8. Reducing Sugars Fermentation to Ethanol

Yeast cells were incubated fed-batch at 27 °C in a liquid yeast medium (g/L: glucose—20, peptone—10, yeast extract—5). To conduct the biocompatibility test, cells were collected and centrifuged for 5 min at 3000 rpm until the stationary phase was achieved (for example, 24-h incubation for *S. cerevisiae*). Then, the supernatant was removed, and the pellet was diluted to a desired cell concentration with pre-sterilized solution (g/L: (NH_4_)_2_SO_4_—5.0, KH_2_PO_4_—0.8, MgSO_4_·7H_2_O—0.5, NH_4_Cl—0.5, K_2_HPO_4_—0.15).

The biocompatibility of the yeasts strains in pairs and in consortia was determined by the direct co-culture method on a solid nutrient medium (N. A. Glushanova). A suspension of the culture grown in a liquid nutrient medium (g/L: glucose—20, peptone—10, yeast extract—5) with optical density (OD, absorbance) up to 0.10–0.11 was applied to a solid nutrient medium with a 3 mm bacteriological loop. After the drop was absorbed, a drop of another test culture was applied to the surface of the same medium. When spreading, the latter was covered by half with the first drop. Petri dishes were incubated for 24 h at a temperature of 27 °C.

To carry out fermentation, a 5 vol.% yeast suspension (with a concentration of 0.60–0.61 optical density) was obtained using the procedure described above and added to hydrolyzates. Fermentation was carried out stationary in a thermostat at 27 °C for 150 h (6.25 days) with a periodic sampling of the supernatant (1 mL) for the GC analysis.

#### 2.3.9. GC Analysis for Ethanol Content Measure

##### Sample Preparation

An aliquot (2 mL) of the fermentation suspension was taken and ethanol was extracted with diethyl ether. A sample was transferred into a 15 mL vial and diluted with 2 mL of diethyl ether. The vial was placed on an orbital shaker for mixing for 30 min. Then, the sample was centrifuged at 3900 rpm for 10 min. The upper organic layer was taken into a flask for the subsequent evaporation of diethyl ether. Then, 1 mL of ethyl acetate was added to the sample, previously evaporated not to a dry condition, and the resulting solution was placed in a vial and injected in the GC.

The ethanol concentration in the reaction mixture was measured after cellulose fermentation by GC-FID analysis [17]. A 1 mL aliquot of the reaction mixture was placed on an SPE column and eluted with 5 mL of dichloromethane. An amount of 1 μL of the solution in dichloromethane after elution was injected in the GC-FID (gas chromatography with flame ionization detector). GC analysis was performed using the GC 7890 B system by Agilent Technologies. GC parameters were as follows: helium as carrier, flow rate 1 mL/min, temperature programming from 50 °C/4 min to 150 °C/5 min (10 °C/min), injector temperature of 180 °C, splitless ratio of 50:1, and volume of injection of 1 μL. FID parameters: H2 flow—30 mL/min, air flow—300 mL/min, and detector temperature of 150 °C. The identification of the alcohol content was carried out using a calibration curve of a mixture of standards, which consisted of ethanol, propanol, and butanol (see Supplementary Materials). The measurements were carried out in three parallels.

## 3. Results

### 3.1. Delignification Efficiency

**Figure 1:**
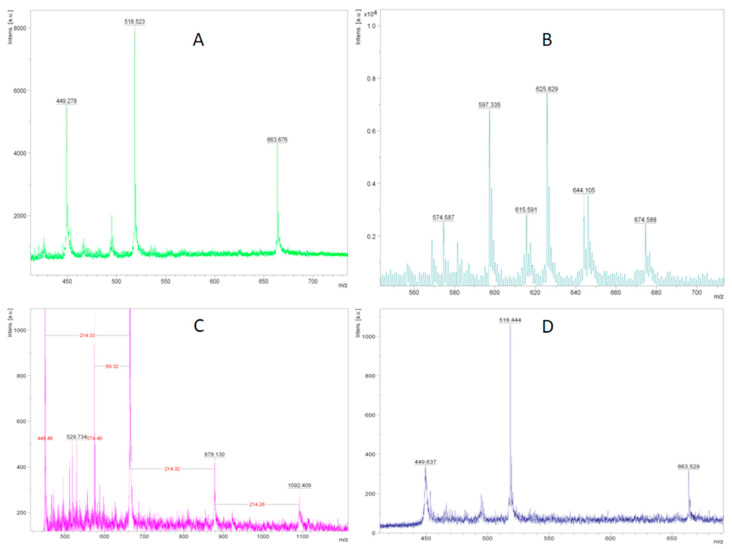
MALDI-TOF (matrix-assisted laser desorption/ionization with time of flight mass spectrometry) spectra of lignin extracts after pretreatment of Miscanthus plant by different methods: (**A**) Method A—NaOH; (**B**) method B—CH_3_COOH/H_2_O_2_; (**C**) method C—benzoic acid/H_2_O_2_; (**D**) method D—TFA/H_2_O_2_.

The MALDI-TOF analysis of the lignin after extraction by different methods showed a different degree of destruction as specified in Figure 1.

**Figure 2:**
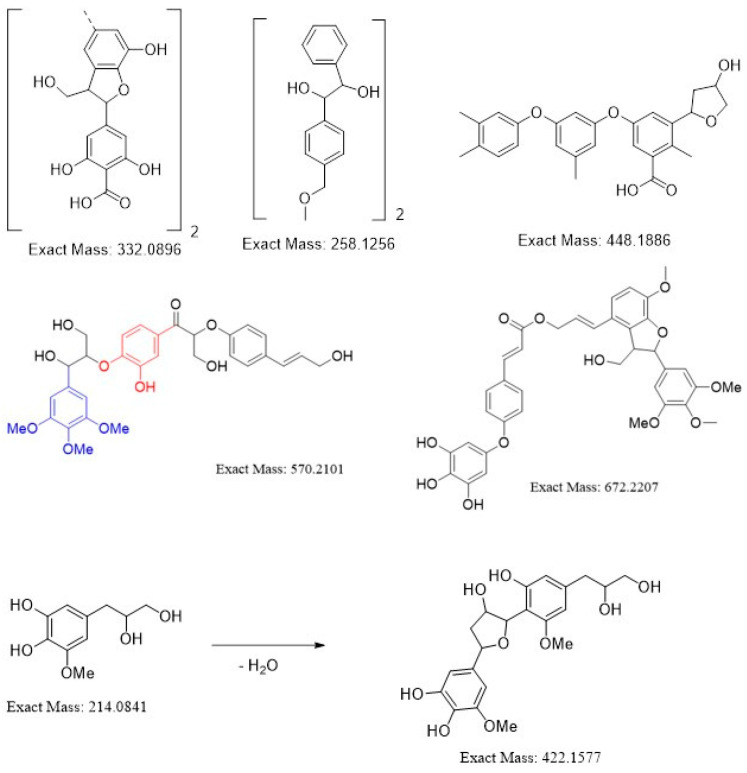
Structures of the molecular fragments of lignin obtained after pretreatment of *Miscanthus* plant with TFA/H_2_O_2_.

As can be seen from Figure 1, method D proved to be the most attractive approach. Indeed, no high molecular weight fractions were observed in the MALDI-TOF spectra. The cellulose obtained via method D possessed a high degree of whiteness, as well as a low lignin content. Therefore, in further experiments, we used the TFA/H_2_O_2_ system for the delignification. Furthermore, the analysis of the spectra thus obtained allowed us to determine the structures of the yielding lignin fragments, as shown in Figure 2.

The quality and yield of cellulose in the case of methods A and D are presented in Table 1. Cellulose yield was determined as the ratio of the mass of cellulose obtained to the mass of the feedstock. Crystallinity was measured by Jayme and Knolle [18].

Noticeably, the degree of cellulose crystallinity reached 67% when method D was employed, and the target product fraction amounted to 54% of the feedstock by weight. A comparison of the quality results of the obtained pulp allows us to conclude that all four methods give an acceptable quality. The method allows for accelerated delignification, which is its advantage.

### 3.2. Saccharification of Cellulose

The following potential microorganisms were chosen for the cellulose fermentation step: *K. Marxianus* Y-2039, *K. Marxianus* Y-2137, *S. Stipites* Y-3263, *S. Stipites* Y-3264, *P. Tannophilus* Y-2246, *P. Tannophilus* Y-3269, *P. Tannophilus* Y-3270, *S. cerevisiae Q* Y-4245, *S. cerevisiae M* Y-4242, *S. cerevisiae W* Y-4246, *S. cerevisiae N* Y-4243, *S. ludwigii 8* Y-2012, *Z. Rouxii* Y-4659, *S. Bacillaris* Y-4015, *L. Thermotolerans* Y-4532, and *T. Delbrueckii* Y-1539.

Initially, all the microorganisms listed above have been tested for their mutual biocompatibility in pairs by means of the co-cultivation method. The study revealed nine strains to be biocompatible, as demonstrated in Table 2. These strains were subsequently screened to develop a microbial consortium for the glucose fermentation in the Miscanthus-derived cellulose hydrolyzate. The selection of the strains to a consortium was carried out in a pseudo-random manner, since all presented microorganisms are potentially capable of the fermentation of sugars and have similar cultivation conditions. For this purpose, three drops of the culture suspension were sequentially applied to Petri dishes.

The most viable consortia corresponded to numbers 8 and 9. Interestingly, the significant growth enhancement was observed for these frontrunner strains, showing their pronounced synergy. These consortia were therefore chosen for further investigation.

Besides that, the *S. cerevisiae M*-mediated fermentation of cellulose, leading to the formation of RS (reducing sugars) was investigated. The results of these efforts are summarized in Table 3.

The performed study allowed us to conclude that the optimal time for cellulose fermentation under these conditions is 90 h.

### 3.3. Fermentation of Reducing Sugars to Ethanol

In furthering the research, the fermentation of the obtained reducing sugars solution was studied. In this regard, results of the yields of ethanol formed under the action of various types of microorganisms were measured. The kinetic parameters were determined according to Equation (1) for the fermentation rate constant and are presented in Table 2.

(1) $$K_{f}=\frac{2.303}{\tau_{f}}\cdot lg\frac{S_{0}}{S}$$

where *K_f_* is the fermentation rate constant, h^−1^, *τ_f_* is the time from the start of fermentation, h, and *S*_0_, *S* are the RS concentrations at the start of fermentation and during fermentation *τ_f_*, mg/mL.

In order to estimate the rate of conversion of sugars, we used *K* (Table 4 and Table 5).

We found that ethanol production was most efficient when employing the consortium of microorganisms consisting of *Saccharomyces cerevisiae M Y-4242*, *Pachysolen tannophilus Y-3269*, and *Scheffersomyces stipitis Y-3264*. The study of the resulting mixture revealed a high degree of selectivity of the fermentation process, since the total content of methanol, butanol, and propanol isomers constituted less than 0.5% of the total alcohol yield and the main component of the alcohol mixture was ethanol.

The samples taken for ethanol detection were as follows:

Sample 1—hydrolyzate after enzymatic hydrolysis of *Aspergillus niger* F-1270, subjected to fermentation by Consortium #8, cultivated under stirred conditions in a shaker incubator at 100 rpm at a temperature of 27 °C.

Sample 2—hydrolyzate after enzymatic hydrolysis of *Coprinus delicatulus* F-248, subjected to fermentation by Consortium #8, cultivated under stirred conditions in a shaker incubator at 100 rpm at a temperature of 27 °C.

Sample 3—hydrolyzate after enzymatic hydrolysis by consortium of *Aspergillus niger* F-1270 and *Coprinus delicatulus* F-248, subjected to fermentation by Consortium #8, cultivated under stirred conditions in a shaker incubator at 100 rpm at a temperature of 27 °C.

Taking into account the initial RS concentration expressed as glucose in the initial enzyme hydrolyzate, i.e., for Sample 1 (15.44 mg/mL or 85.704 mmol/L), Sample 2 (15.40 mg/mL or 85.481 mmol/L), and Sample 3 (13.465 mg/mL or 74.741 mmol/L), the ethanol yields were calculated (Table 6).

In general, we found that the cellulose obtained from Miscanthus using trifluoroacetic acid can be processed into bioethanol using microorganisms. The results are consistent with previously published works. The innovation of the study lies in the fact that we offer a protocol for processing Miscanthus growing in Russia. The results obtained indicate the possibility of obtaining cellulose from this plant by standard methods. To obtain bioethanol from cellulose, we selected a consortium of microorganisms that are available for industrial use in Russia, which does not require the organization of logistics for the delivery of microorganisms.

## 4. Discussion

In our study, we have identified an effective method of Miscanthus plant delignification in a medium of trifluoroacetic acid and hydrogen peroxide, leading to cellulose with a high degree of crystallinity. The fragmentation of the resulting lignin turned out to be the highest among the tested approaches. Thus, the obtained cellulose can be subsequently hydrolyzed to reducing sugars under the action of a fungi consortium of *Aspergillus niger* F-1270 *+ Coprinus delicatulus* F-248. Meanwhile, the resulting mixture of reducing sugars can be processed into ethanol when subject to fermentation by the yeasts consortium of *Saccharomyces cerevisiae* M Y-4242/*Pachysolen tannophilus* Y-3269/ *Scheffersomyces stipitis* Y-3264.

Regarding the rate of fermentation (Table 2 and Table 3), it should be noted, that even though the K values were higher in the case of Consortium #9 (*Pachysolen tannophilus* Y-3269/*Lachancea thermotolerans* Y-4532/*Kluyveromyces lactis* Y-2039), the initial glucose concentration was significantly (approximately twice) higher than that for Consortium #8 (*Saccharomyces cerevisiae* M Y-4242/*Pachysolen tannophilus* Y-3269/*Scheffersomyces stipitis* Y-3264). Therefore, similar RS concentrations obtained by four–five days with both consortia indicate Consortium #8 to be more efficient. Since the RS concentrations obtained after six days equalized and slightly decreased, when using both consortia, it can be suggested that five days was the optimal fermentation time for these systems.

Importantly, the highest ethanol yield (3.429%) was achieved for Sample 1 under the following conditions: hydrolyzate yielding from enzymatic hydrolysis by *Aspergillus niger* F-1270 was subjected to fermentation by Consortium #8 under stirred conditions in a shaker incubator at 100 rpm at a temperature of 27 °C.

To sum up, we have developed a novel protocol for the complex processing of Miscanthus plants into ethanol, which involves delignification with trifluoroacetic acid and hydrogen peroxide, followed by cellulose fermentation into ethanol by yeast consortia: *Aspergillus niger* F-1270 *+Coprinus delicatulus* F-248, and *Saccharomyces cerevisiae* M Y-4242/*Pachysolen tannophilus* Y-3269/ *Scheffersomyces stipitis* Y-3264. The suggested approach allows the convenient production of a high-quality product and the yields are in line with the recently reported analogs designed for other plants [19]. The composition of the occurring sugars remains to be investigated. Furthermore, the efforts for the identification of the enzymes involved in cellulose fermentation are currently being undertaken and will be reported in due course.

## Figures and Tables

**Table 1:** The quality and yield of cellulose.

Parameter	Method A	Method B	Method C	Method D
Average cellulose yield (%)	48.07 ± 1.25	52.47 ± 1.25	45.08 ± 2.34	62.54 ± 1.25
Crystallinity (%)	54.2	58.7	55.2	61.4
Lignin content (%) by (ISO/DIS 21436)	1.40 ± 0.25	0.8 ± 0.25	1.1 ± 0.25	less than 0.5

**Table 2:** Combinations of yeast strains consortia evaluated for their biocompatibility.

No.	Microorganism	Strain	Y-4242	Y-3269	Y-4532	Y-4015	Y-4659	Y-4245	Y-2246	Y-3264	Y-2039
1	*S. cerevisiae M*	Y-4242	1							8	
2	*P. tannophilus*	Y-3269									9
3	*L. thermotolerans*	Y-4532		2							
4	*S. bacillaris*	Y-4015			3						
5	*Z. rouxii*	Y-4659				4					
6	*S. cerevisiae Q*	Y-4245					5				
7	*P. tannophilus*	Y-2246						6			
8	*S. stipites*	Y-3264							7		
9	*K. marxianus*	Y-2039									

**Table 3:** Yield of reducing sugars during cellulose fermentation by a yeast consortium of *Saccharomyces cerevisiae* M Y-4242/*Pachysolen tannophilus* Y-3269/ *Scheffersomyces stipitis* Y-3264.

Fermentation of Cellulose by *Aspergillus niger* F-1270 *+Coprinus delicatulus* F-248				
Fermentation Time, h	Probe #	Absorbance	C Reducing Sugars mg/mL	Cellulose Conversion %
12	0	0.052	4.59 ± 0.20	18 ± 1.25
24	1	0.062	5.58 ± 0.20	23 ± 1.25
48	2	0.068	7.03 ± 0.20	34 ± 1.25
72	3	0.101	9.45 ± 0.20	64 ± 1.25
96	4	0.138	13.21 ± 0.20	72 ± 1.25
120	5	0.240	13.46 ± 0.20	75 ± 1.25

**Table 4:** Ethanol yield after fermentation of a reducing sugar solution by Consortium #8.

Fermentation_*Saccharomyces cerevisiae* M Y-4242/*Pachysolen tannophilus* Y- 3269/*Scheffersomyces stipitis* Y-3264 (Consortium #8)						
Time, h	Probe #	Absorbance	C Reducing Sugars mg/mL	*K_f_* 10^3^ h^−1^	Reducing Sugars Conversion, %	Ethanol Concentration by GC-FID g/L
0	0	0.232	22.41 ± 0.20	-	-	-
24	1	0.213	20.53 ± 0.20	3.653	8.39 ± 0.50	1.02 ± 0.20
48	2	0.118	16.69 ± 0.20	6.142	25.53 ± 0.50	2.12 ± 0.20
72	3	0.079	14.53 ± 0.20	6.018	35.16 ± 0.50	3.02 ± 0.20
96	4	0.071	6.85 ± 0.20	12.349	69.44 ± 0.50	7.04 ± 0.20
120	5	0.064	5.78 ± 0.20	11.294	74.21 ± 0.50	8.59 ± 0.20
144	6	0.051	4.49 ± 0.20	1.116	79.95 ± 0.50	9.23 ± 0.20

**Table 5:** Ethanol yield after fermentation of a reducing sugar solution by Consortium #9.

Fermentation_ *Pachysolen tannophilus* Y-3269 /*Lachancea thermotolerans* Y-4532/*Kluyveromyces lactis* Y-2039 (Consortium #9)						
Time, h	Probe #	Absorbance	C Reducing Sugars mg/mL	*K_f_* 10^3^ h^−1^	Reducing Sugars Conversion, %	Ethanol g/L by GC-FID
0	0	0.260	12.59 ± 0.20	-	-	-
24	1	0.201	9.67 ± 0.20	10.996	23.20 ± 0.50	1.38 ± 0.20
48	2	0.190	8.95 ± 0.20	7.116	28.93 ± 0.50	1.81 ± 0.20
72	3	0.125	5.91 ± 0.20	10.508	53.06 ± 0.50	3.36 ± 0.20
96	4	0.110	5.16 ± 0.20	9.280	58.96 ± 0.50	3.75 ± 0.20
120	5	0.109	5.12 ± 0.20	7.504	59.35 ± 0.50	3.82 ± 0.20
144	6	0.089	4.13 ± 0.20	7.746	67.21 ± 0.50	4.27 ± 0.20

**Table 6:** Ethanol yields after fermentation.

Probe Name	Ethanol Concentration by GC-FID g/L	Ethanol Yield, %, per g RS in Hydrolyzate	Ethanol Yield, %, per g Biomass
Sample 1	5.58 ± 0.20	71 ± 1.5	3.40 ± 0.5
Sample 2	5.28 ± 0.20	67 ± 1.5	3.30 ± 0.5
Sample 3	5.09 ± 0.20	74 ± 1.5	3.40 ± 0.5

## Supplementary Materials

The following are available online at https://www.mdpi.com/2306-5354/7/2/61/s1. Electronic supporting information for this article contains a description of general methods and additional experimental data.
