# Supplementary material for: The Process of Producing Bioethanol from Delignified Cellulose Isolated from Plants of the Miscanthus Genus
Source: Bioengineering (Basel). 2020 Jun 21;7(2):61. doi: 10.3390/bioengineering7020061 (PMC7355652; doi:10.3390/bioengineering7020061)
Supplement: Supplementary file 1 [file bioengineering-07-00061-s001.pdf]

Supporting Information for

The Process of Producing Bioethanol from Delignified Cellulose Isolated from Plants of the Miscanthus Genus

Olga Kriger [1], Ekatrina Budenkova [1], Olga Babich [1,2], Stanislav Suhih [1,2], Nikolay Patyukov [1], Yakov Masyutin [1], Vyacheslav Dolganuk [2], Evgenii Chupakhin [1,*]

Contents:

Experimental procedures and data pp. 2-7

Materials

All analytical grade chemicals (like cellobiose, glucose, xylose, arabinose, furfural, hydroxymethyl furfural, acetic acid, propanol, butanol, ethanol, methanol, sodium acetate) and ligno-cellulolytic enzymes cellulose (Celluclast 1.5 L), β-glucosidase (i.e., BGL or Novozym 188 in this study), xylanase were procured from Sigma Aldrich, German.

Enzyme Hydrolysis of Cellulose

Batch hydrolysis was carried out in sodium acetate buffer (50 mM, pH 4.8) at 2% (w/w) solids loading. The reaction mixtures (1 mL) were mechanically shaken in an orbital shaker (Combi-D24 hybridization incubator) at 50 °C. Enzymatic cocktail (prepare by analogue [1]) protein content: Cellulase from *Aspergillus niger* 800 units/g and Xylanase from *Asp. oryzae* 2500 unis/g in ratio 4:3. was added at 15 or 30 mg cocktail/g cellulose biomass treatment by GP1. Over a period of 72 h, samples were taken and heated at 100 °C for 10 min, and centrifuged at 4,000 rpm for 15 min at room temperature. The supernatants were collected for quantification of glucose released, and the residual hydrolysis materials were retained for fiber analysis. All of the experiments were performed in triplicate.

General Procedure for Determination of Reducing Sugars after Fermentation of Cellulose [2]

The method is based on reduction of 3,5-dinitrosalicylic acid (yellow) with reducing sugars to 3-amino-5-nitrosalicylic (yellow-orange). The resulting 3-amino-5-nitrosalicylic acid is determined spectrophotometrically at a wavelength of 530 nm.

Calculation of the reducing sugars (RS) concentration is carried out according to the calibration plot (Figure S1).

The concentration of reducing sugars in the solution should be within the range from 0.2 to 2 g/L, taking into account the sensitivity of the method.

The method consists of two main stages.

a) Preparation of 3,5-dinitrosalicylic-acid-based reagent;
b) Spectrophotometric detection of reducing sugars (RS) concentration expressed as a glucose concentration.

A. 16.05 g of sodium hydroxide was dissolved in 150 cm^3 of distilled water to prepare 10.7 wt% aqueous solution. The resulting solution was cooled to room temperature.

10.0 g of 3,5-dinitrosalicylic acid (DNS) and 400 cm^3 of distilled water were transferred into a 1 L beaker to prepare 1.0 wt% aqueous solution. The solution was stirred for 25–30 min at room temperature. Then 150 cm^3 of the above aqueous solution of sodium hydroxide was added gradually, with constant stirring. The color of the solution obtained was changed from light yellow to bright yellow.

A beaker with the solution obtained was transferred into a water bath with a temperature set of 47 ± 1 °C and then 300 g on dry basis of sodium potassium tartrate are gradually added in small portions. Stirring was being continued at the above temperature until the reagent was completely dissolved.

The solution was cooled with cold water to a room temperature, transferred to a 1 L volumetric flask, brought to the mark with distilled water, and filtered through a funnel with a glass filter if necessary. The reagent obtained should have a bright yellow color, without a red tint.

The shelf life of the above solution in a dark bottle at room temperature is no more than 6 months.

In case of precipitation, the solution must be filtered through a funnel with a glass filter.

B. 1 cm^3 of the test solution filtered if necessary and 2 cm^3 of the DNS reagent obtained was pipetted in 25 cm^3 volumetric flasks, and mixed up rapidly. A control sample was prepared simultaneously: 2 cm^3 of the DNS reagent was added to 1 cm^3 of distilled water, and mixed up.

The flasks are placed in a boiling water bath and boiled for 5 min with an accuracy measured by a stopwatch or hourglass. The flasks were cooled to a room temperature with cold water, adjusted to the mark with distilled water, mixed thoroughly, and the absorbancy of the solutions was measured by means of Schimadzu UV-3600 dual-beam spectrophotometer at a wavelength of 530 nm in cuvettes with a 10 mm thick light-absorbing layer.

The working area of the calibration plot lies within the absorbancy from 0.15 to 1.5. The absolute error of this method is 0.01725 g/L. The relative error is 3.45%.

The convergence of measurements is 0.0019 g/L.

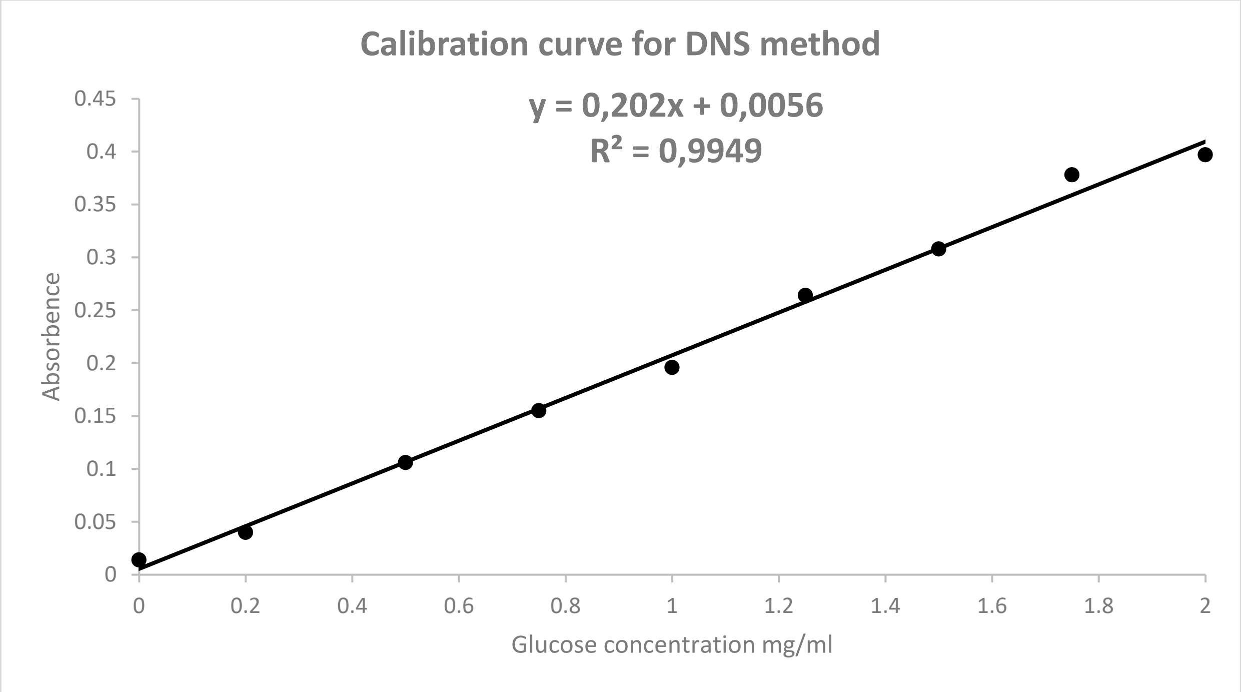

Figure S1. Calibration curve for determination the reducing sugar by DNS method

Hydrolysis of Miscanthus-Derived Cellulose by Microorganisms Analogue to [3]

Strains of microscopic fungi *Aspergillus niger* F-1270 and *Coprinus delicatulus* F-248 were cultivated on solid nutrient medium (g/L: maltose - 30, peptone - 1, agar - 20). After the second passage, a suspension in a culture medium was prepared from one colony of the strain (g/L: maltose - 30, peptone - 1). To carry out enzymatic hydrolysis,

A suspension of microorganisms (5 vol.%) with a concentration of 0.60–0.61 OD was inoculated to a substrate sample in an acetate buffer solution (0.1 g/mL, pH = 5.0). If the suspension was inoculated to consortium of microorganisms, the total volume of the inoculum was divided equally between the components of the consortium. Enzymatic hydrolysis was carried out in an incubator shaker at 30 °C and 100 rpm for 5 days. Samples for photometric analysis were taken once a day at regular intervals.

Ethanol Fermentation

Commercial Baker's yeast (*Saccharomyces cerevisiae*) obtained from a local market, was used for the ethanol fermentation. Inoculum was prepared by transferring yeast cells (1.0 g/100 mL) into 250 mL flasks containing 50.0 mL of culture medium containing 10.0 g/L yeast extract, 20.0 g/L peptone, and 20.0 g/L glucose and subsequently incubating at 30.0 °C for 24.0 h. This was used to inoculate the fermentation medium. Cellulosic hydrolyzate, obtained from enzymatic hydrolysis, was supplemented with 1.0 g/L yeast extract, 2.0 g/L $(NH_4)_2SO_4$ and 1.0 g/L of $MgSO_4$. The inoculum-to-solution ratio of 1:10 was used for fermentation purposes. Samples for glucose and ethanol analysis were taken at the beginning and end of a 24.0 h fermentation process.

Cellulose Fermentation to Ethanol [4,5]

Yeast cells in a suitable growth phase were harvested and centrifuged for 5 min at 3000 rpm. The supernatant was removed, the pellet was diluted with pre-sterilized solution (g/L: $(NH_4)_2SO_4$ - 5.0, KH_2PO_4 - 0.8, $MgSO_4{\cdot}7H_2O$ - 0.5, NH_4Cl - 0.5, K_2HPO_4 - 0.15) to achieve a suitable cell concentration (0.60 - 0.61 OD). A suspension of microorganisms (5 vol.%) was added to the medium (250 µL per 5 mL). Fermentation was carried out stationary in a thermostat at 27 °C for 150 h (6.25 days) with supernatant taken periodically and then filtered. RS were determined spectrophotometrically expressed as a glucose concentration, using DNS reagent method. Ethanol was detected by means of GC.

K – fermentation rate constant

$$K_f = \frac{2.303}{\tau_f} \cdot lg\frac{S_0}{S}$$

where K_f – fermentation rate constant, h^{-1};

τ_f – time from the start of fermentation, h;

S_0, S – RS concentrations at the start of fermentation and during fermentation τ_f, mg/mL.
In order to estimate the rate of conversion of sugars we used K.
Saccharomyces cerevisiae M Y-4242/*Pachysolen tannophilus* Y- 3269/*Scheffersomyces stipitis* Y-3264

GC Analysis for Ethanol Content Measure

A sample (2 mL) after fermentation with the ethanol was extracted with diethyl ether. A sample was transferred into a 15 mL vial, then 2 mL of diethyl ether was poured into it. The vial was placed on an orbital shaker for mixing for 30 min. Then the sample was centrifuged at 3900 rpm for 10 min. The upper organic layer was taken into a flask for subsequent evaporation of diethyl ether. Then 1 mL of ethyl acetate was added to a sample, previously evaporated not to dry condition, the resulting solution was placed in a vial and injected in GC.

GC Analysis

Ethanol content in reaction mixture after cellulose fermentation was measure by GC-FID analysis [4]. 1 mL aliquot of reaction mixture was place on SPE column and elution 5 mL dichloromethane. 1 μL solution dichloromethane after elution was injection in GC. GC analysis performed on a GC 7890 B system from Agilent Technologies. GC parameter: helium as carrier with flow rate 1 mL/min and temperature programming from 50 °C/4 min to 150 °C/5 min (10 °C/min), temperature of injector 180 °C, splitless ratio 50:1, volume of injection 1 μL. FID parameter: H_2 flow - 30 mL/min, air flow – 300 mL/min, detector temperature 150 °C. Identification of the substances was carried out by mix of alcoholic standards. Calibration plot for ethanol content was made within the range from 0,03 to 10 vol.%

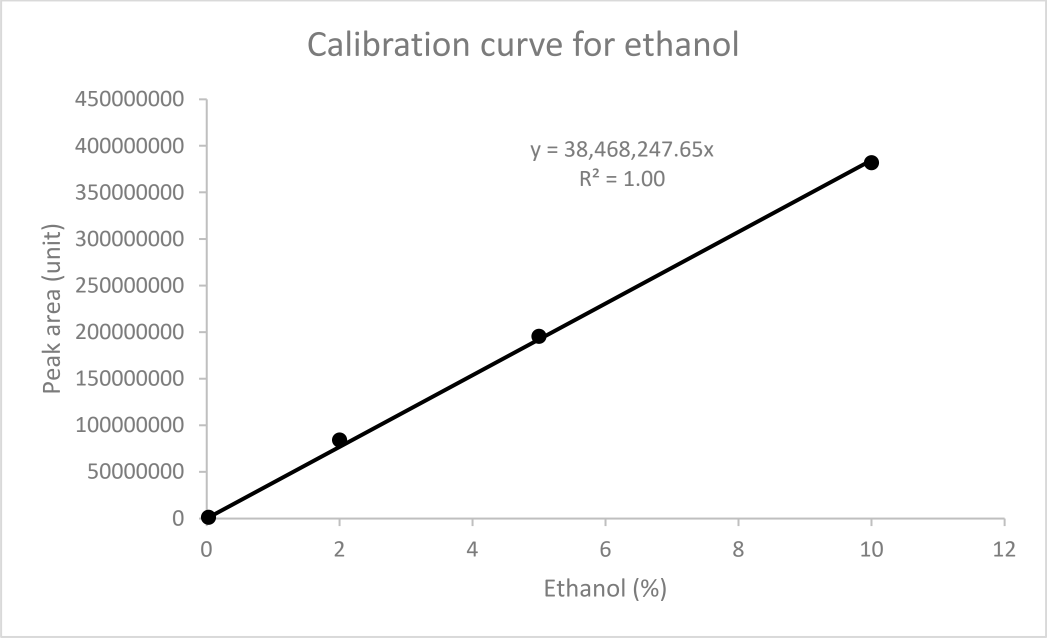

Figure S2. Calibration curve for ethanol determination by GC-FID.

Biocompatibility Test for Artificial Consortia of Microorganisms

1. Enzymatic Hydrolysis by Means of Fungi

The biocompatibility of microscopic fungi strains *Aspergillus niger* F-1270 and *Coprinus delicatulus* F-248 was carried out by the direct co-cultivation method [3–7].

To conduct the biocompatibility test of the strains investigated, suspensions in culture medium (g/L: maltose – 30, peptone – 1) with an optical density of 0.10–0.11 were made.

A suspension drop of one strain was applied with 3 mm bacteriological loop to the surface of a solid nutrient medium. The second drop of different test strain were appled next to the dried previous one in such a way that they overlap each other by half. Petri dishes were incubated for 24 hours at a temperature of 25 °C.

The test was carried out in triplicate. There was also the control biocompatibility test, when two drops of the same strain were applied to the surface.

The results were interpreted as follows:

- The growth retardation of one strain consider as antagonism.
- The spot of one culture, shifting to the surface, regardless of application sequence consider as weak antagonism.
- Increased growth of the studied strains or merging of spots consider as biocompatibility of cultures.

2. Fermentation by means of yeasts [9]

Yeast cells were incubated fed-batch at 27 °C in liquid yeast medium (g/L: glucose - 20, peptone - 10, yeast extract - 5). To conduct a biocompatibility test, cells had been collected and centrifuged for 5 minutes at 3000 rpm before the stationary phase was achieved (for example, 24-hour incubation for *S. cerevisiae*). Then supernatant was removed, the pellet was diluted to a desired cell concentration with pre-sterilized solution (g/L: $(NH_4)_2SO_4$ - 5.0, KH_2PO_4 - 0.8, $MgSO_4 \cdot 7H_2O$ - 0.5, NH_4Cl - 0.5, K_2HPO_4 - 0.15). The biocompatibility of the strains tested was determined by the method of the direct co-culture on a solid nutrient medium. A suspension of the culture grown in a liquid nutrient medium (g/L: glucose - 20, peptone - 10, yeast extract - 5) with OD up to 0.10–0.11 was applied to a solid nutrient medium with a 3 mm bacteriological loop. After the drop was absorbed, a drop of another test culture was applied to the surface of the same medium. When spreading, the latter covered half of the first drop. Petri dishes were incubated for 24 hours at a temperature of 27 °C. The cultures were cultivated, competing with each other in the superimposed areas. Two drops of the same culture, applied according the above-described technique, were used as a control test. The results were interpreted as follows.

- The growth retardation of one strain consider as antagonism.
- The spot of one culture, shifting to the surface, regardless of application sequence consider as weak antagonism.
- Increased growth of the studied strains or merging of spots consider as biocompatibility of cultures.

1. Agrawal, R.; Semwal, S.; Kumar, R.; Mathur, A.; Gupta, R. P.; Tuli, D. K.; Satlewal, A. Synergistic Enzyme Cocktail to Enhance Hydrolysis of Steam Exploded Wheat Straw at Pilot Scale. *Front. Energy Res.* **2018**, *6*, 122. https://doi.org/10.3389/fenrg.2018.00122.
2. Zhao, C.; Deng, L.; Fang, H. Mixed Culture of Recombinant Trichoderma Reesei and Aspergillus Niger for Cellulase Production to Increase the Cellulose Degrading Capability. *Biomass and Bioenergy* **2018**, *112*, 93–98. https://doi.org/https://doi.org/10.1016/j.biombioe.2018.03.001.
3. Karagöz, P.; Özkan, M. Ethanol Production from Wheat Straw by Saccharomyces Cerevisiae and Scheffersomyces Stipitis Co-Culture in Batch and Continuous System. *Bioresour. Technol.* **2014**, *158*, 286–293. https://doi.org/https://doi.org/10.1016/j.biortech.2014.02.022.
4. Esteves, M.; Barbosa, C.; Vasconcelos, I.; Tavares, M. J.; Mendes-Faia, A.; Mira, N. P.; Mendes-Ferreira, A. Characterizing the Potential of the Non-Conventional Yeast Saccharomycodes Ludwigii UTAD17 in Winemaking. *Microorganisms* **2019**, *7* (11), 478. https://doi.org/10.3390/microorganisms7110478.
5. Salimi, F.; Mahadevan, R. Characterizing Metabolic Interactions in a Clostridial Co-Culture for Consolidated Bioprocessing. *BMC Biotechnol.* **2013**, *13* (1), 95. https://doi.org/10.1186/1472-6750-13-95.
6. Begunova, A. V; Rozhkova, I. V; Zvereva, E. A.; Glazunova, O. A.; Fedorova, T. V. Lactic and Propionic Acid Bacteria: The Formation of a Community for the Production of Functional Products with Bifidogenic and Hypotensive Properties. *Appl. Biochem. Microbiol.* **2019**, *55* (6), 660–669. https://doi.org/10.1134/S0003683819060048.
7. Wang, Z.; Yan, M.; Chen, X.; Li, D.; Qin, L.; Li, Z.; Yao, J.; Liang, X. Mixed Culture of Saccharomyces Cerevisiae and Acetobacter Pasteurianus for Acetic Acid Production. *Biochem. Eng. J.* **2013**, *79*, 41–45. https://doi.org/https://doi.org/10.1016/j.bej.2013.06.019.
